# Reproductive performance and fertility traits in Madrasin cattle: The influence of *growth differentiation factor-9* gene expression on reproductive tract size, cervical mucus characteristics, and fertility rate

**DOI:** 10.14202/vetworld.2025.1799-1806

**Published:** 2025-07-08

**Authors:** Budi Utomo, Rimayanti Rimayanti, Rochmah Kurnijasanti, Nurhusien Yimer Degu, Athhar Manabi Diansyah, Muhammad Fajar Amrullah

**Affiliations:** 1 Division of Veterinary Reproduction, Faculty of Veterinary Medicine, Universitas Airlangga, Surabaya 60115, East Jawa, Indonesia; 2 Division of Basic Veterinary, Faculty of Veterinary Medicine, Universitas Airlangga, Surabaya 60115, East Jawa, Indonesia; 3 School of Veterinary Medicine, IMU University, 57000, Bukit Jalil, Federal Teritory of Kuala Lumpur, Malaysia; 4 Department of Animal Production, Faculty of Animal Science, Hasanuddin University, Makassar 90245, South Sulawesi, Indonesia

**Keywords:** artificial insemination, cervical mucus, fertility marker, *GDF-9* gene, Madrasin cattle, reproductive performance

## Abstract

**Background and Aim:**

The *growth differentiation factor-9* (*GDF-9*) gene is recognized as a critical regulator of ovarian function and fertility in cattle. However, its role in crossbred populations, particularly Madrasin cattle (Madura × Simmental cross), remains underexplored. Understanding the genetic underpinnings of fertility traits in this crossbreed could provide valuable insights for improving reproductive efficiency in Indonesia’s livestock sector. This study aimed to investigate the relationship between *GDF-9* gene expression and reproductive traits, specifically reproductive tract size (RTS), cervical mucus characteristics, and fertility rate, in Madrasin cows.

**Materials and Methods:**

A total of 20 Madrasin cows (aged 2–5 years) were evaluated. *GDF-9* expression levels were assessed through polymerase chain reaction, and samples were categorized into “Strong Expression” (G1) and “Dimmed Expression” (G2) groups based on amplicon band intensity. Reproductive tract dimensions were recorded through rectal palpation, while cervical mucus quality was analyzed using five parameters: appearance, viscosity, spinnbarkeit, pH, and fern pattern. Fertility was determined by the non-return rate (NRR) following artificial insemination. Statistical analysis was conducted using analysis of variance with a significance threshold of p < 0.05.

**Results:**

Cows in the strong *GDF-9* expression group exhibited significantly larger uterine horn diameters and greater ovarian height than the dimmed group (p < 0.05). Cervical mucus from G1 animals displayed higher scores in appearance and spinnbarkeit, although only appearance was statistically significant. NRR was notably higher in G1 (p < 0.05), suggesting enhanced fertility in cows with elevated *GDF-9* expression.

**Conclusion:**

*GDF-9* gene expression in Madrasin cattle is positively associated with RTS and fertility-related cervical mucus characteristics. These findings suggest that *GDF-9* could serve as a promising genetic marker for improving fertility and breeding outcomes in crossbred cattle populations. However, the study’s limited sample size and exclusion of environmental factors warrant further investigation to validate the utility of *GDF-9* in broader genetic selection strategies.

## INTRODUCTION

Crossbreeding has become a pivotal strategy in Indonesia for improving cattle productivity, which is vital for maintaining the national meat supply, particularly among smallholder farmers who dominate the livestock sector [1, 2]. To achieve this, Indonesian farmers cross local breeds such as the heat-tolerant Madura cattle (*Bos javanicus*) with *Bos taurus* breeds (e.g., Simmental), aiming to combine the environmental resilience of *Bos indicus* with the superior growth and meat quality traits of *B. taurus* [3, 4]. Crossbred animals such as Madrasin cattle (Madura × Limousin) have been developed to enhance productivity and production efficiency, making them well-suited to Indonesia’s diverse agro-climatic conditions [5].

Despite their economic significance, the reproductive traits of crossbred cattle have not been thoroughly investigated, thus limiting their optimized use in breeding and meat production systems. Reproductive performance in cattle is determined by a complex interplay of genetic, environmental, and management factors [6, 7]. Among these, reproductive tract size (RTS) and cervical mucus quality serve as well-established indicators of bovine fertility [8, 9]. Parameters, such as uterine horn diameter and ovarian dimensions, provide critical insight into reproductive health and are strongly associated with successful breeding outcomes [10]. Similarly, cervical mucus assessment, including param-eters such as viscosity, spinnbarkeit, pH, and fern patt-ern, is crucial for determining the optimal timing for insemination, given its close relationship with estrus and fertility [9]. These phenotypic markers are integral to improving the success rate of artificial insemination (AI), a widely implemented genetic enhancement met-hod in Indonesian cattle production.

Beyond phenotypic traits, the identification of genetic markers linked to fertility has become increasingly prominent in reproductive biotechnology [11]. The *growth differentiation factor-9* (*GDF-9*) gene, a key regulator of ovarian function, plays an essential role in follicular development, oocyte maturation, and overall reproductive success in bovines [12]. Notably, two single-nucleotide polymorphisms (SNPs) within intron 1A485T and A625T have been reported to influence the number of transferable embryos and total ova in Chinese Holstein cows [13]. Moreover, five non-synonymous mutations in the *GDF-9* gene have been detected in Peranakan Ongole and Belgian Blue cattle, resulting in amino acid substitutions that may affect the functional properties of the *GDF-9* protein [14]. Such polymorphisms have been linked to variation in fertility and follicle development, as demonstrated in Awassi sheep, where specific SNPs were associated with increased follicle size and oocyte yield [15, 16].

While crossbreeding programs such as the development of Madrasin cattle (Madura × Limousin) have significantly contributed to the productivity and adaptability of livestock in Indonesia, the reproductive performance of these crossbreds remains inadequately characterized. Most prior studies have focused on phenotypic performance metrics such as growth rate, carcass quality, and feed conversion efficiency, with limited attention given to fertility-related parameters. Moreover, although RTS and cervical mucus quality are well-established predictors of fertility in purebred cattle, their predictive value in crossbred populations, especially in relation to genetic markers, has not been sufficiently explored. In particular, the *GDF-9* gene, a key regulator of ovarian folliculogenesis and oocyte maturation, has been studied in various breeds for its association with superovulation and embryo yield. However, no published studies have yet investigated the expression pattern of the *GDF-9* gene and its relationship to reproductive traits in Madrasin cattle. This lack of genetic and physiological data hampers the implementation of marker-assisted selection strat-egies in crossbreeding programs targeting reproductive efficiency.

The present study aims to evaluate the expression of the *GDF-9* gene in Madrasin cattle and to elucidate its association with critical reproductive parameters. Specifically, the study investigates the relationship between *GDF-9* gene expression levels and RTS (including uterine horn and ovarian dimensions), cervical mucus characteristics (appearance, viscosity, spinnbarkeit, pH, and fern pattern), and fertility outcome measured by the non-return rate (NRR) following AI. By integrating molecular, anatomical, and physiological data, this study seeks to determine whether *GDF-9* expression can serve as a viable genetic marker for reproductive performance in Madrasin cattle. The findings are expected to support the development of evidence-based selection criteria in genetic improvement programs for enhanced reproductive efficiency in Indonesia’s crossbred cattle population.

## MATERIALS AND METHODS

### Ethical approval

All procedures involving animals were reviewed and approved by the Animal Ethics Committee of the National Research and Innovation Agency (Approval No. 065/KE.02/SK/10/2022).

### Study period and location

The study was conducted from May to December 2024. The animals were sourced from local farmers in Bangkalan Regency, East Java. The samples were processed at the Faculty of Veterinary Medicine, Universitas Airlangga.

### Experimental design

This study involved 20 Madrasin cows aged 2–5 years, obtained from local farmers in East Java, Indonesia. The animals were managed under a semi-extensive production system. All cows were categorized into two experimental groups based on the band intensity of their *GDF-9* gene polymerase chain reaction (PCR) amplicons: Strong Expression and Dimmed Expression (Table 1). The study consisted of three major assessments: reproductive tract measurements, cervical mucus evaluation, and determination of fertility rates.

**Table 1:** The categories of the *GDF-9* gene PCR product amplicons.

Group category	Description
G1 (Strong expression)	The display of the amplicon band shows a very high-intensity band on the gel documentation system. Light intensity categorization into strong (intensity >70)
G2 (Dimmed)	The display of the amplicon band has a very low dim intensity on the gel documentation system. Light intensity categorization into dimmed (intensity <70)

*GDF-9=Growth differentiation factor-9*, PCR=Polymerase chain reaction

### Blood sample collection and DNA extraction

Jugular blood samples (3 mL/cow) were collected using ethylenediaminetetraacetic acid (EDTA)-coated vacuum tubes (Vaculab EDTA K3, OneMed, Surabaya, Indonesia). Samples were immediately stored in an icebox and transported to the laboratory for DNA isolation. Genomic DNA was extracted using the PureLink Genomic DNA mini kit (Invitrogen K-182001, USA) and stored at −20°C until further use [17].

### Primer design and PCR amplification of the GDF-9 gene

*GDF-9* gene expression was assessed using duplex PCR, following the protocol described by Utomo *et al*. [18]. Primers developed by Arta *et al*. [19] were used:

- Forward primer: 5′-GCCCACCCACACACCTAAAGTT TA-3′
- Reverse primer: 5′-GCACACCAACAGCTGAAAGAGG TA-3′

The 25 μL PCR reaction mixture consisted of 12.5 μL GoTaq Green master mix (Promega, USA), 2 μL DNA template, 1 μL of each primer (10 pmol), and 8.5 μL of nuclease-free water. Amplification was performed using a GeneAmp thermal cycler (Applied Biosystems, Thermo Fisher Scientific, MA, USA) with the following cycling conditions:

- Initial denaturation at 95°C for 5 min
- 34 cycles of 95°C for 45 s, 61°C for 45 s, 72°C for 45 s
- Final extension at 72°C for 10 min.

### Visualization and classification of PCR amplicons

PCR products were visualized on 1.5% agarose gels stained with SYBR Green (Invitrogen S7563, Thermo Fisher Scientific, Eugene, USA) and separated using gel electrophoresis as described by Belgania *et al*. [20]. Amplicon sizes were assessed using a 1000-bp molecular marker (Thermo Scientific DNA Ladder). Gel images were captured using a documentation system, and band intensity was quantified using ImageJ software (https://imagej.net/ij/index.html). Bands were classified based on light intensity:

- Strong Expression: Intensity >70
- Dimmed Expression: Intensity <70.

These intensity values were considered proxies for relative gene expression levels [20].

### Assessment of RTS

Reproductive tract measurements were conducted through rectal palpation during estrus, following the method of Yusuf *et al*. [9]. The uterine horn diameter was measured to evaluate uterine size and condition. Ovarian dimensions – height, length, and width – were also recorded to assess overall ovarian development.

### Evaluation of cervical mucus characteristics

Cervical mucus samples were obtained 5–30 min before AI during estrus. Parameters assessed inclu-ded appearance, viscosity, spinnbarkeit, pH, and fern pattern, using the protocol described by Diansyah *et al*. [21]. Each parameter was scored as follows:

- Appearance: 0 (opaque/dirty) to 3 (clear/transparent)
- Viscosity: 0 (highly viscous) to 3 (watery)
- Spinnbarkeit: 0 (<6 cm), 1 (7–12 cm), 2 (13–18 cm), 3 (>19 cm)
- pH (test strip): 0 (pH <7 or >8.6) to 3 (pH 7.6–8.0)
- Fern Pattern: 0 (no crystallization) to 3 (tertiary/quaternary branching).

Cervical mucus collection and evaluation were per-formed by trained inseminators immediately before AI.

### Fertility rate assessment

Fertility outcomes were evaluated *in vivo* using the NRR as a proxy for AI success, based on methods outlined by Diansyah *et al*. [21]. Cows that did not return to estrus within 21 days post-insemination were considered successfully inseminated. External factors, such as insemination timing, semen quality, or environmental stressors, were not controlled for in this study.

### Statistical analysis

All data were analyzed using a one-way analysis of variance to compare reproductive tract measurements, cervical mucus characteristics, and fertility rates between Strong and Dimmed *GDF-9* expression groups. Statistical analyses were performed using Statistical Package for the Social Sciences version 25 (IBM Corp., Chicago, IL, USA). Results were considered statistically significant at p < 0.05.

## RESULTS

### PCR amplification of the *GDF-9* gene in Madrasin cattle

**Figure 1:**
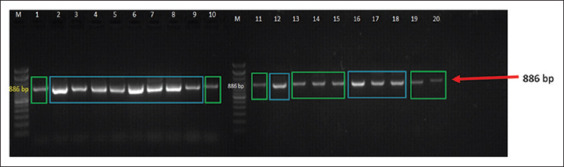
Product size of polymerase chain reaction using primers in the *growth differentiation factor-9* (*GDP-9*) target gene of Madrasin Cattle, Lane M=Marker; Lane 1–20=Sample with amplified *GDF-9* gene DNA fragment. The green squares on the sample bands indicate Strong expression categories, and the blue squares indicate dimmed categories.

PCR successfully amplified an 886-bp fragment of the *GDF-9* gene in all 20 Madrasin cattle samples (Figure 1). Gel electrophoresis revealed a single, distinct DNA band for each sample, with no evidence of non-specific amplification or smearing, indicating both high PCR efficiency and intact genomic DNA. The uniformity and clarity of the bands demonstrated the high specificity of the primer sets used. Based on band intensity, the animals were categorized into two expression groups: Group 1 (Strong Expression, n = 12) and Group 2 (Dimmed Expression, n = 8). The consistent detection of the target amplicon in all samples confirmed the success of the DNA amplification process.

### RTS comparison between expression groups

**Figure 2:**
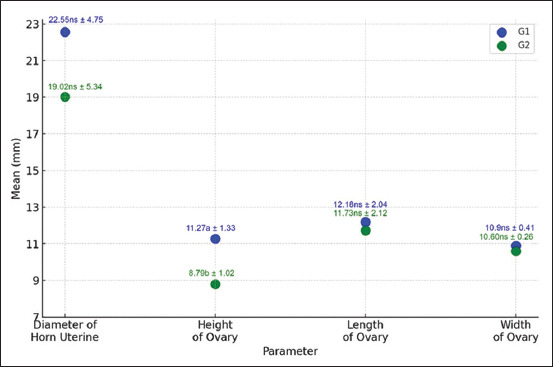
Reproductive tract size in Madrasin cattle; G1=Strong expression; G2=Dimmed expression; (different superscripts (a, b) accompanying the data indicate significance (p < 0.05); ns=non-significant).

As shown in Figure 2, reproductive tract measur-ements differed between Group 1 and Group 2. Notably, the uterine horn diameter was significantly greater in cows from Group 1 compared to those in Group 2 (p < 0.05), suggesting a correlation between *GDF-9* expression and uterine development. Similarly, ovarian height was significantly higher in Group 1 than in Group 2, indicating enhanced ovarian development in animals with stronger gene expression. However, ovarian length and ovarian width did not differ significantly between the two groups, suggesting that not all ovarian dimensions are influenced equally by *GDF-9* expression.

### Evaluation of cervical mucus parameters

**Figure 3:**
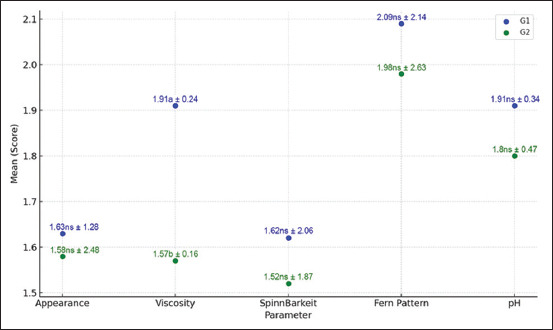
Cervical mucus characteristics in Madrasin cattle; G1=Strong expression; G2=Dimmed expression; (different superscripts [a, b] accompanying the data indicate significance [p < 0.05]; ns=Non-significant).

Figure 3 presents the comparative analysis of cervical mucus characteristics between the two expression groups. In terms of appearance, Group 1 exhibited significantly higher scores, indicating clearer and more transparent mucus, which is typically assoc-iated with higher fertility. While viscosity scores were slightly higher in Group 1, the difference was not statistically significant, suggesting similar mucus consi-stency across both groups. No significant diffe-rences were observed in spinnbarkeit, fern pattern, or pH, indicating that these parameters may not be strongly influenced by variations in *GDF-9* expression.

### Fertility rate based on NRR

**Figure 4:**
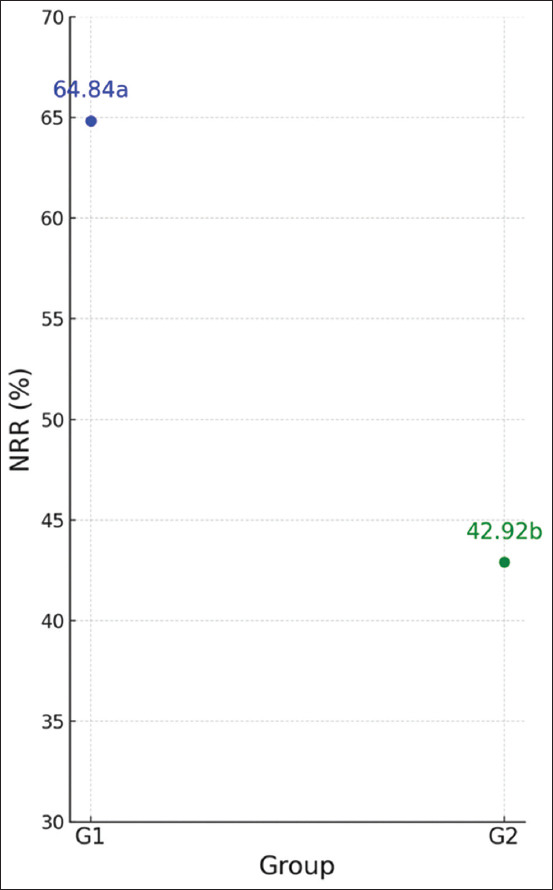
Fertility rate in Madrasin cattle; G1=Strong expression; G2=Dimmed expression; (different letters [a, b] accompanying the data indicate significance [p < 0.05]).

The fertility performance, as measured by the NRR, is illustrated in Figure 4. Group 1 animals demonstrated a significantly higher NRR compared to Group 2 (p < 0.05), indicating enhanced reproductive success in cows with elevated *GDF-9* expression. These findings support the hypothesis that higher *GDF-9* gene expression is positively associated with improved fertility outcomes in Madrasin cattle.

## DISCUSSION

### Crossbreeding strategies and the need for reproductive improvement

The Indonesian livestock sector, particularly in beef production, faces the dual challenge of increasing output while ensuring long-term sustainability [22]. To address this, crossbreeding of *B. javanicus* (Madura cattle), which are well adapted to tropical environments, with *B. taurus* breeds, such as Simmental has become a widely adopted strategy [23]. This hybridization aims to combine the environmental resilience of *B. indicus* with the superior growth rates and meat quality of *B. taurus* [24]. The resulting Madrasin cattle represent a promising genetic resource, offering both productivity and adaptability for national meat supply chains [25]. However, despite their economic importance, the reproductive performance of Madrasin cattle has not been thoroughly characterized, posing a major barrier to the effectiveness of genetic improvement programs [26].

### The role of *GDF-9* in crossbred fertility: Rationale and objectives

Reproductive efficiency in cattle is influenced by a complex interaction of genetic, physiological, and environmental factors [27, 28]. Among these, the *GDF-9* gene, a key regulator of ovarian folliculogenesis and oocyte maturation, holds substantial promise as a molecular marker of fertility [29]. This study aimed to investigate the relationship between *GDF-9* gene expression and fertility-related traits in Madrasin cattle, including RTS, cervical mucus characteristics, and NRR following AI.

### *GDF-9* expression patterns and group stratification

Using PCR, *GDF-9* gene expression was assessed by analyzing amplicon intensity in 20 Madrasin cows. As shown in Figure 1, animals were categorized into two expression groups: Group 1 (Strong Expression) and Group 2 (Dimmed Expression). The high specificity and uniform amplification efficiency observed confirmed both primer fidelity and DNA integrity [30]. Stronger band intensities in Group 1 suggest elevated *GDF-9* expression, which is closely associated with granulosa cell function, follicular growth, and oocyte maturation – mechanisms previously described by Wang *et al*. [31] and Xie *et al*. [32]. This variability in gene expression among individuals may underlie key differences in reproductive performance and offers potential for use in selective breeding programs [33].

### *GDF-9* expression and RTS

RTS is a well-established physiological indicator of fertility. In this study, RTS was evaluated by measuring uterine horn diameter and ovarian dimensions through rectal palpation. As shown in Figure 2, cows in Group 1 demonstrated significantly greater uterine horn diam-eter and ovarian height compared to Group 2. These findings are consistent with previous studies, which indicate that larger reproductive organs are associated with better embryo implantation and higher conception rates [34, 35]. The results suggest that elevated *GDF-9* expression may enhance ovarian function and foll-icular development, as further supported by Haimon *et al*. [36]. The observed association between *GDF-9* expression and RTS thus reinforces its relevance as a potential biomarker for reproductive efficiency in cross-bred cattle.

### Cervical mucus quality as a fertility indicator

Cervical mucus plays a pivotal role in determining the success of AI, as it influences sperm transport and fertilization. This study found that Group 1 cows exhibited more favorable cervical mucus characteristics, including increased spinnbarkeit, clearer appearance, optimal pH, and more distinct fern patterns (Figure 3). While viscosity and pH differences were not statistically significant, the superior mucus appearance and trend toward higher spinnbarkeit suggest a better estrus environment in cows with stronger *GDF-9* expression. These observations align with findings by Cordova-Gomez *et al*. [37] and Maru and Kumar [38], who reported that fertility-associated genes, including *GDF-9*, correlate with improved cervical mucus profiles. Indirect modulation through estrogen may be a plaus-ible mechanism, as elevated estrogen levels, linked to follicular development, are known to enhance mucus clarity and elasticity [39, 40].

### Fertility outcomes correlate with *GDF-9* expression

Fertility was assessed through the NRR, a standard measure of insemination success. As depicted in Figure 4, Group 1 cows had significantly higher NRR compared to Group 2, supporting the hypothesis that elevated *GDF-9* expression enhances fertility. These results align with prior research, which has shown that high *GDF-9* expression enhances oocyte quality, embryo development, and pregnancy rates in cattle [41, 42]. The data suggest that *GDF-9* expression may serve as a predictive marker of reproductive success and could be utilized in breeding programs to reduce calving intervals and increase conception rates.

### Implications for genetic selection and breeding programs

The findings from this study underscore the utility of *GDF-9* gene expression as a potential molecular marker for fertility in Madrasin cattle. Strong *GDF-9* expression was associated with larger reproductive tracts, improved cervical mucus traits, and significantly higher fertility outcomes. These characteristics position *GDF-9* as a promising candidate for incorporation into marker-assisted selection strategies aimed at enhancing reproductive efficiency and genetic gain in Indonesian crossbreeding programs. The integration of *GDF-9* analysis in selection protocols could improve the identification of high-fertility animals, thereby promoting sustainable cattle production.

### Study limitations and future directions

Despite the promising associations observed, this study presents several limitations. The relatively small sample size (n = 20) restricts the generalizability of the results, and larger cohort studies are needed to validate the findings. In addition, the analysis focused solely on genetic factors, without accounting for environmental influences such as nutrition, housing, and stress, all of which could modulate gene expression and reproductive performance. Another limitation is the cross-sectional nature of the gene expression analysis, which was conducted at a single time point. Longitudinal studies examining temporal patterns of gene expression and multigenerational fertility outcomes would offer dee-per insights. Finally, while PCR effectively quantified expression levels, more advanced techniques such as RNA sequencing and transcriptomic profiling could elucidate the regulatory mechanisms underlying *GDF-9* function.

## CONCLUSION

This study provides compelling evidence that *GDF-9* gene expression is positively associated with reproductive efficiency in Madrasin cattle, a crossbred population critical to Indonesia’s livestock productivity. Cows exhibiting strong *GDF-9* expression demonstrated significantly larger reproductive tract dimensions, clearer and more favorable cervical mucus characteristics, and higher fertility rates as indicated by elevated NRRs. These findings suggest that *GDF-9* plays a pivotal role in regulating reproductive physiology and may serve as a reliable genetic marker for selecting high-fertility animals.

The practical implications of these results are substantial for breeding programs in tropical and crossbreeding systems. Incorporating *GDF-9* gene expression profiling into selection protocols could enhance reproductive performance, reduce calving intervals, and increase AI success rates – key priorities for sustainable cattle production. This approach offers a cost-effective, molecular-level tool to complement phenotypic assessments, thereby improving the preci-sion of fertility-based selection in crossbred herds.

A major strength of the study lies in its integrated evaluation of molecular, anatomical, and physiological parameters, providing a multidimensional under-stand-ing of fertility in Madrasin cattle. The use of direct *in vivo* fertility outcomes (NRR) strengthens the practical relevance of the findings. Furthermore, the study intro-duces a novel application of *GDF-9* expression analysis in a crossbred population where such genetic associations have previously been underexplored.

In conclusion, the *GDF-9* gene emerges as a promising biomarker for enhancing reproductive management in Indonesian cattle systems. However, given the study’s limited sample size and exclusion of environmental variables, future research should employ larger, more diverse populations and longitudinal designs. Incorporating transcriptomic and hormonal profiling may further elucidate the mechanistic role of *GDF-9* in bovine reproduction. Ultimately, the strategic integration of genetic markers, such as *GDF-9*, into national breeding frameworks could accelerate genetic gains and ensure reproductive sustainability in tropical livestock systems.

## AUTHORS’ CONTRIBUTIONS

BU, RR, RK, and NYD: Conceived, designed, and coordinated the study. BU and NYD: Designed data collection tools. RR, RK, and MFA: Supervised field sampling, data collection, laboratory work, and data entry. BU and AMD: Statistical analysis. BU, AMD, and MFA: Statistical analysis and interpretation. All authors have read and approved the final version of the manuscript.
